# Ensemble Approach for Detection of Depression Using EEG Features

**DOI:** 10.3390/e24020211

**Published:** 2022-01-28

**Authors:** Egils Avots, Klāvs Jermakovs, Maie Bachmann, Laura Päeske, Cagri Ozcinar, Gholamreza Anbarjafari

**Affiliations:** 1 iCV Lab, Institute of Technology, University of Tartu, 51009 Tartu, Estonia; egils.avots@ut.ee (E.A.); klavs.jermakovs@ut.ee (K.J.); chagri.ozchinar@ut.ee (C.O.); 2 Biosignal Processing Laboratory, Tallinn University of Technology, 19086 Tallinn, Estonia; maie.bachmann@taltech.ee (M.B.); laura.paeske@taltech.ee (L.P.); 3 PwC Advisory, 00180 Helsinki, Finland; 4 Faculty of Egineering, Hasan Kalyoncu University, 27000 Gaziantep, Turkey

**Keywords:** depression, electroencephalogram (EEG), feature extraction and selection, machine learning, ensemble learning

## Abstract

Depression is a public health issue that severely affects one’s well being and can cause negative social and economic effects to society. To raise awareness of these problems, this research aims at determining whether the long-lasting effects of depression can be determined from electroencephalographic (EEG) signals. The article contains an accuracy comparison for SVM, LDA, NB, kNN, and D3 binary classifiers, which were trained using linear (relative band power, alpha power variability, spectral asymmetry index) and nonlinear (Higuchi fractal dimension, Lempel–Ziv complexity, detrended fluctuation analysis) EEG features. The age- and gender-matched dataset consisted of 10 healthy subjects and 10 subjects diagnosed with depression at some point in their lifetime. Most of the proposed feature selection and classifier combinations achieved accuracy in the range of 80% to 95%, and all the models were evaluated using a 10-fold cross-validation. The results showed that the motioned EEG features used in classifying ongoing depression also work for classifying the long-lasting effects of depression.

## 1. Introduction

Depression is a major public health problem, creating a significant burden throughout the world. The World Health Organization (WHO) has predicted depression to be one of the most common causes of work disability [1]. According to disability-adjusted life-years or illness, depression ranks first in many European countries [2,3]. The largest aggregate study of the prevalence of mental disorders in the European population shows that clinically significant depression has been experienced by an average of 6.9% of the population in a 12 mo period [2].

Depression is a mental disorder characterised by a pathologically low mood with a negative, pessimistic assessment of oneself, one’s position in the surrounding reality, and one’s future. Depression causes emotional, psychological, and physical suffering, which lead to a decrease in the patient’s quality of life, family, work, and social adaptation, and often to disability. However, the worst consequence of depression is the increased risk of committing suicide.

Currently, the most common way to diagnose depression is an interview conducted by a medical professional. In many cases, the interview is accompanied with a clinical questionnaire assessed by a medical doctor such as the Hamilton Depression Rating Scale (HAM-D), the self-reported Emotional State Questionnaire (EST-Q) [4], or Mini-Mental State Examination (MMSE) [5] to establish the diagnostic criteria. Other questionnaires, such as the Beck Depression Inventory (BDI) [6] and the Hamilton Depression Rating Scale (HDRS) [7], are also used for screening purposes.

Besides subjective clinical questionnaires, the brain activity of the patients can be monitored objectively by applying various imaging modalities such as computed tomography (CT), functional magnetic resonance imaging (fMRI), and electroencephalogram (EEG). Out of these techniques, EEG stands out as the simplest and most cost effective. Hence, detecting mental states and disorders by using various EEG feature representations, such as methods based on fast Fourier transform (FFT), discrete wavelet transform (DWT), power spectral analysis (PSA), and others [8,9,10,11,12,13], is an actively researched field showing promising results. Various advanced machine learning algorithms have been utilised in order to analyse different modalities of such data in order to introduce automated assessment of depression [13,14,15,16,17,18,19].

This paper reports the classification results obtained by using various linear and nonlinear features and provides a general insight into the feature calculation. The main contribution of the paper is the feature selection and best-performing feature combinations. This article also describes several classifier configuration that improve the classification accuracy.

## 2. Related Work

According to de Aguiar Neto et al. [20], absolute and relative band powers and various other linear and also nonlinear features described in this section have been recognised as promising biomarkers for characterizing a depressed brain.

The absolute band power (ABP) and relative band power (RBP) of EEG signals have been analysed with separate three-way multivariate analysis of variance (MANOVA) and showed that the RBP was greater in depressed patients than in controls at all electrode locations and increased ABP for some of the electrode locations [21].

The use of alpha power variability (APV) and relative gamma power (RGP) was proposed by Bachmann et al. [8]. While APV indicates the power and frequency variations in the alpha band, RGP characterises the high-frequency components. The differences between the depressed and control groups appeared statistically significant in a number of EEG channels, leading to a linear regression classification accuracy of 81%.

The spectral asymmetry index (SASI) indicates the relative asymmetry between higher and lower frequency bands. According to Hinrikus et al. [22], SASI values differed significantly in all channels between healthy and depressed patients. Single EEG channel analysis has already shown positive results in the detection of depression [8,23].

The nonlinear Higuchi’s fractal dimension (HFD) calculates the fractal dimension of a signal in the time domain [24]. Bachmann et al. [25] applied the HFD method for EEG signals and evaluated this using Student’s T-test for two-tailed distributions with two-sample unequal variance, to find if a statistical difference existed between depressed and healthy subjects. The alterations were statistically significant in all the EEG channels and indicated 94% of the subjects as depressive in the depressive group, while HFD indicated 76% of the subjects as non-depressive in the control group.

The nonlinear Lempel–Ziv complexity (LZC), introduced by Lempel and Ziv [26], measures the complexity of a signal and has been successfully used on EEG signals for the detection of different mental states [27,28]. EEG data from severe Alzheimer’s disease patients showed a loss of complexity over a wide range of time scales, indicating a destruction of nonlinear structures in brain dynamics [29,30,31].

Detrended fluctuation analysis (DFA) [32], which indicates long-time correlations of the signal, was applied to evaluate EEG signals and revealed a statistically significant difference between healthy and depressive subjects [33]. In addition, linear discriminant analysis (LDA) reached a classification accuracy of 70.6%, and by combining DFA and the SASI, classification accuracy increased to 91.2% [23].

A comprehensive study by Bachmann et al. [8] showed the diagnostic potential for linear (SASI, APV, RGP) and nonlinear (HFD, DFA, LZC) features to classify depression. Single-channel classification with logistic regression achieved an accuracy of 81% using APV or RGP measures. The combination of two linear measures, the SASI and RGP, reached an accuracy of 88%, and by combining linear and nonlinear measures, a classification accuracy of 92% was achieved [8].

## 3. Experimental Setup

### 3.1. EEG Recording Procedure

**Figure 1:**
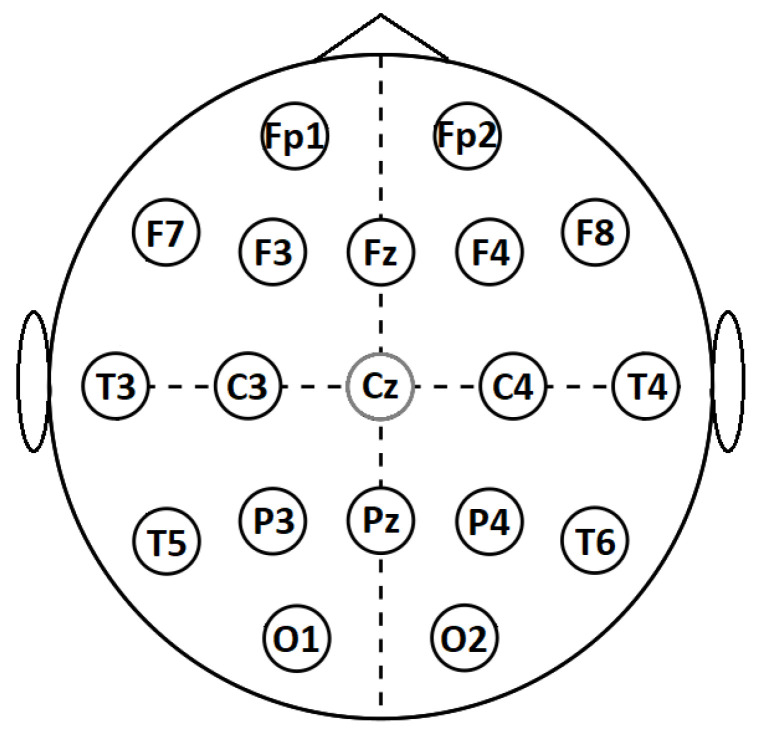
International 10–20 system for EEG recording.

The Cadwell Easy II EEG (Kennewick, WA, USA) measurement equipment was used for EEG recordings with 18 channels (reference Cz), which were placed on the subject’s head according to the international 10–20 electrode position classification system, as shown in Figure 1. During the recordings, the subjects were lying in a relaxed position with their eyes closed. EEG signals within the frequency band of 3–48 Hz were used for further processing. The sample rate was kept at 400 Hz for linear methods, while the downsampled signals with a sample rate of 200 Hz were used for nonlinear methods, due to the high computational load. The 20 min-long EEG recording was segmented into 10 s segments, and an experienced EEG specialist marked the first 30 artefact-free segments (5 min in total) by visual inspection, for the subsequent feature calculation.

The gathering of questionnaires and EEG recordings were carried out by Tallinn University of Technology (TalTech), in accordance with the Declaration of Helsinki, and the process was formally approved by the Tallinn Medical Research Ethics Committee. All participants signed a written informed consent. The dataset itself was provided to the authors by Tallinn University of Technology under a legal agreement for research purposes. (Information about obtaining the dataset can be requested by contacting M. Bachmann at maie.bachmann@taltech.ee.)

### 3.2. Dataset

The recorded dataset consisted of the EEG signals from 20 subjects, who were selected for further analyses from 55 subjects, who regularly visited the occupational health doctor. The dataset consisted of 14 females and 6 males within the age range of 24–60 y. Half of the subjects selected had been diagnosed with depression at some point in their lives (referred to as depressed subjects for simplicity), while the healthy control group had never had a depression diagnosis. In addition, the healthy control group was chosen considering their low HAM-D and EST-Q scores, to ensure they did not exhibit any signs of depression or other mental disorders (see Table 1). All subjects were gender matched, and the subject age for healthy controls was chosen to be as close as possible to the age of depressed subjects.

### 3.3. Hamilton Depression Rating Scale

The HAM-D is the most widely used clinician-administered depression assessment scale. Although the rating scale has been criticised for use in clinical practice, in this study, it was used as additional information for selecting healthy subjects. In situations where more than one healthy subject was a match candidate for a depressive subject, the one with the lowest HAM-D score was chosen. The mean HAM-D score among the healthy subjects was 3.1, where the scores of 0–7 indicate no depression and a mean score of 9.3 for the depressive subjects corresponds to mild depression.

### 3.4. Emotional State Questionnaire

The Emotional State Questionnaire (EST-Q) [34] was originally compiled for use by the lecturers of the psychiatric clinic of the University of Tartu in Estonia. The self-assessed questionnaire consists of 28 statements assessing the major depressive and anxiety disorders and their associated symptoms during the last month. The questionnaire consists of 3 basic scales and 3 additional scales. Major scales include the depression (DEP), general anxiety (AUR), and panic agoraphobia subscales (PAF). Additional subscales include social anxiety (SAR), asthenia (AST), and insomnia, which was not used. The scale’s total score can be used as an overall indicator of the severity of emotional symptoms. The EST-Q was used in the current study for selecting healthy subjects. The subscale values of all the selected subjects were below the threshold for the given condition, except for 2 healthy subjects, whose asthenia subscale was greater than 6. Other threshold values can be found in Table 1. If the scale value is greater than the listed threshold, then the subject has the given condition.

## 4. Features

EEG brain signals are nonlinear by nature and linked to particular brain activity, which can be analysed through various linear and nonlinear signal-processing methods.

### 4.1. Linear Features

#### 4.1.1. Relative Band Power

One of the most widely used methods to analyse EEG signals is to decompose the signal into functionally distinct frequency bands, such as delta (1–4 Hz), theta (4–8 Hz), alpha (8–12 Hz), beta (12–30 Hz), and gamma (30–45 Hz). In the current study, this was achieved by first calculating the power spectral density of the EEG signal by Welch’s method, as done by Bachmann et al. 2018. EEG powers in the theta, alpha, beta, and gamma frequency bands were computed by integrating the power spectral density at the frequencies within the boundary frequencies of the EEG spectral bands. Relative band powers (T_rbp_, A_rbp_, B_rbp_, G_rbp_) are expressed as the power in the specific EEG frequency band as a percentage of the total power of the signal.

#### 4.1.2. Alpha Power Variability

The alpha band signal (8–12 Hz) was obtained by a pass-band filter. Next, the APV was calculated for the artefact-free 10 s segments in three steps. First, the alpha band signal power in time window *T* for N=4000 samples was calculated as:

(1) $$W_{i}=\frac{1}{N}\sum_{r=1}^{N}{\left[V\left(r\right)\right]}^{2}$$

where V(r) is the amplitude of the alpha band signal in a sample *r* and *N* is the number of samples in the time window *T*. Afterwards, APV was calculated as:

(2) $$APV=\frac{\sigma }{W_{0}}$$

where $W_{0}$ is the value of alpha band power averaged over 5 min and σ is the standard deviation of those segments.

#### 4.1.3. Spectral Asymmetry Index

The SASI evaluates the power in higher and lower frequencies and was calculated as the relative difference between the higher and the lower EEG frequency band power. The balance of the powers characterises the EEG spectral asymmetry [22]. Powers in the frequency bands were calculated as:

(3) $$P_{\delta mn}=\sum_{f_{i}=F_{c}-6}^{f_{i}=F_{c}-2}S_{mn}$$

and:

(4) $$P_{\beta mn}=\sum_{f_{i}=F_{c}+2}^{f_{i}=F_{c}+26}S_{mn}$$

where $F_{c}$ is the central frequency of the EEG spectrum maximum in the alpha band and was calculated for each person individually. The SASI in channel *m* for a subject *n* was calculated as:

(5) $$SASI_{mn}=\frac{P_{\beta mn}-P_{\delta mn}}{P_{\beta mn}+P_{\delta mn}}\;.$$

### 4.2. Nonlinear Features

Nonlinear methods are used to capture the chaotic behaviour in EEG signals, which occurs due to the underlying physiological activity occurring in the brain [35]. To describe the brain activity of the subjects, we used the Higuchi fractal dimension (HFD), Lempel–Ziv complexity (LZC), and detrended fluctuation analysis (DFA).

#### 4.2.1. Higuchi Fractal Dimension

The fractal dimension provides a measure of the complexity of time series, such as EEG, and describes the fractal dimension of time series signals. The values of the HFD for each electrode were calculated according to Higuchi [24] with the parameter $k_{max}=8$.

#### 4.2.2. Lempel–Ziv Complexity

The complexity of the signal can be quantified by the LZC [36], describing the spatio-temporal activity patterns in high-dimensional nonlinear systems. This can reveal the regularity and randomness in EEG signals. For LZC calculation, each signal segment was converted into a binary sequence s(n) as follows,

(6) $$s\left(n\right)=\left\{\begin{array}{cc} 1, & \mathrm{if}\;x(n)>m\\ 0, & \mathrm{if}\;x(n)\leq m \end{array}\right.$$

where x(n) is the signal segment, *n* is the segment’s sample index from 1 to *N* (segment length), and *m* is the threshold value. The binary sequence s(n) was scanned from left to right counting the number of different patterns. The complexity value c(n) was increased every time a new pattern was encountered. LZC values were calculated as follows:

(7) $$C\left(N\right)=\frac{c(N)}{b(N)}$$

where b(N) is the upper bound of c(n):

(8) $$\lim_{n\to \infty }c\left(n\right)=b\left(N\right)=\frac{N}{log_{a}N}$$

which was used to normalise LZC values to avoid variations in segment length.

#### 4.2.3. Detrended Fluctuation Analysis

DFA is applied to evaluate the presence and persistence of long-range correlations in time in EEG signals. It has been discovered that the resting EEG of healthy subjects exhibits persistent long-range correlation over time [33]. DFA was calculated in the time domain according to the steps described by Peng et al. [32].

## 5. Methodology

The calculated features are represented as 1D vectors constructed from 9 feature types; T_rbp_ (theta), A_rbp_ (alpha), B_rbp_ (beta), G_rbp_ (gamma), APV, SASI, HFD, LZC, and DFA for 18 electrodes; FP1, FP2, F7, F3, FZ, F4, F8, T3, C3, C4, T4, T5, P3, PZ, P4, T6, O1, and O2 (as shown in Figure 1), resulting in 162 unique features. When using the feature evaluation methods, the number of used features can be reduced and concatenated together with other feature types to build more diverse feature sets.

**Figure 2:**
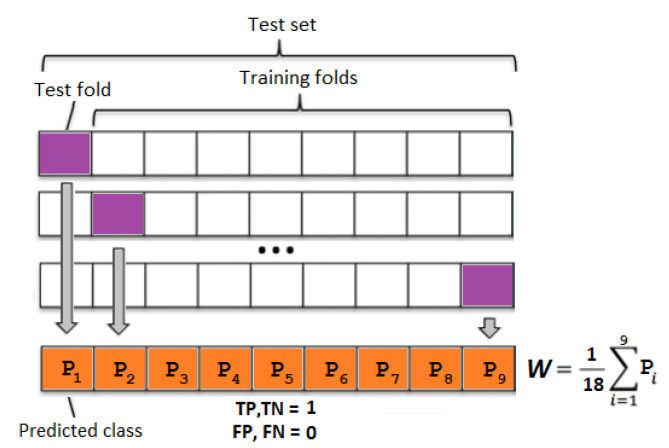
Weight *W* calculation for an ensemble classifier using training data. Each block contains one healthy and depressed subject. TP—true positive, TN—true negative, FP—false positive, and FN—false negative.

All methods were evaluated using a 10-fold cross-validation; in addition, to keep the training data as balanced as possible, each fold had an equal number of healthy and depressed subjects. In the case of predictions for the weighted and boosted ensemble, the training set in each fold underwent an additional 9 iteration procedures (see Figure 2), to obtain prediction results for all samples in the training fold. Afterwards, the weights *W* of the classifier votes were fit according to the results in the training set. Similarly, AdaBoost used predicted class results from the training set to calculate weights for each of the classifiers in the ensemble.

### 5.1. Feature Selection

It is known that cognitive disorders can introduce observable change in measured EEG recordings. Depending on the feature calculations used, each brain region might have a statistically significant difference when compared to cognitively normal patients’ brains. Therefore, to select the most relevant electrode locations, we used feature subset selection methods that were applied in a preprocessing step before machine learning algorithms were applied. In particular, we used the F-test, which is widely used for showing a statistical significance between two classes, and ReliefF, which is a rank-based feature selector.

#### 5.1.1. Univariate Feature Ranking Using F-Tests

The univariate feature ranking algorithm helps to understand the significance of each feature by examining the importance of each predictor individually using an F-test. Each F-test tests the hypothesis that the response values grouped by predictor variable values are drawn from populations with the same mean against the alternative hypothesis, such that the population means are different [37].

#### 5.1.2. ReliefF

The base algorithm Relief, created by Kira and Rendell [38], is an inductive learning system that was initially developed for classifying binary problems using discrete and numerical features. The algorithm penalises the predictors that give different values to neighbours of the same class and rewards predictors that give different values to neighbours of different classes. ReliefF, which is an extended version of Relief algorithm, was developed by Kononenko et al. [39], by proposing the L1 distance for finding near-hit and near-miss instances.

### 5.2. Machine Learning Algorithms

The supervised learning algorithms used in this study have been widely used in various EEG classification tasks according to survey papers published by Lakshmi et al. [40] and other articles, which describe the use of the following algorithms for binary classification:

- Support vector machine (SVM) [41] with the radial basis function (RBF) kernel;
- Linear discriminant analysis (LDA) [42] with the diagonal covariance matrix for each class;
- Naive Bayes (NB) [43];
- K-nearest neighbours (kNN) [44] with 4 neighbours;
- Decision tree (D3) [43].

In addition to individually evaluating the results for the listed classifiers and feature types, an ensemble approach was also implemented, where classifiers trained on all 9 feature types vote to predict the class label.

#### Ensemble Methods

The implemented ensemble [45] votes were weighted according to majority voting, where all weights are equal, and weighted voting, where weights are set according to classifier test set accuracy, which was obtained by the procedure shown in Figure 2. The ensemble assigns Label to a given sample according to the following equation:

(9) $$y=\sum_{n=1}^{m}w_{i}d_{i}$$

where *m* indicates the number of classifiers, $w_{i}$ is the classifier weight, and $d_{i}$ is the classifier decision [1=depressed, −1=healthy]. The class label is decided as follows,

(10) $$Label=\left\{\begin{array}{cc} 1, & \mathrm{if}\;y>0\\ -1, & \mathrm{otherwise}. \end{array}\right.$$

As a third ensemble method, we chose adaptive boosting (AdaBoost) [46], to see if it was possible to find a more optimal weight combination, in comparison to the majority and weighted voting. The aim of AdaBoost is to convert a set of weak classifiers into a strong classifier.

## 6. Results and Discussion

The baseline accuracy was established by individually evaluating all the feature types. In Table 2, the results for classifiers reached acceptable accuracy, where the HFD and LZC reached above 80% with at least one of the classifiers. For other feature types, selecting all electrodes from a feature type did not guarantee the best classification results. For some of the feature types, it is shown that only a few electrodes provided statistically relevant information and the remaining electrodes could be considered as not relevant or redundant [8].

A brute-force approach can be used to check all feature combinations to find which feature sets perform better than others, but this would be a time-consuming process. Therefore, the most relevant features were determined according to feature ranking provided by the F-tests and ReliefF algorithm.

**Figure 3:**
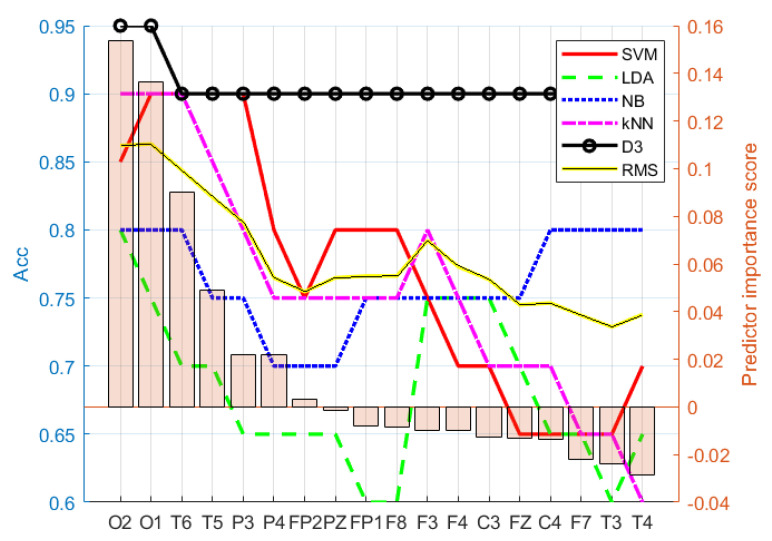
Electrode importance ranking for B_rbp_ according to ReliefF. Feature set {O2,O1} was chosen as the best option for the B_rbp_ selected (ranked) feature set, as the RMS value was highest at electrode O1.

The selected feature evaluation started with the most relevant feature, and in each iteration, the next-less-relevant feature was added to the feature set used in classification. The ranking of the features was provided by the feature-selection algorithms. Each iteration underwent 10-fold cross-validation. The most optimal feature set was selected according to the highest root-mean-squared (RMS) value calculated from the accuracy of all five classifiers for each feature type. Figure 3 shows an example of feature selection according to the described procedure, where electrodes {O2, O1} were selected as the best option for the B_rbp_ feature type, as the highest RMS value was at the O1 electrode. Similarly, the procedure was repeated for all feature types to obtain the best-performing features shown in Table 3. As a limitation, all proposed feature combinations had to be classified; therefore, it can be considered as a computationally heavy process when EEG with more electrodes or large datasets are used.

Compared to the baseline results (Table 2) and selected feature classification results from Table 4 and Table 5, it can be observed that on average, the selected features based on the F-test ranking outperformed the baseline results, and ReliefF had the best overall classification results. In addition, to reduce the effect of subject order in the dataset, the obtained classification results represent the mean results of 100 iterations where the subject location in the training and testing set was randomised.

A more robust solution can be achieved using an ensemble approach where many weak classifiers contribute to the predicted class by voting. Each result shown in Table 6 was the result of combining nine classifiers of the same type. The features used in each feature type were selected according to Table 3. The ensemble approach further improved the results when F-tests and ReliefF feature selection algorithms were used. On average, the ReliefF classification results outperformed ensembles whose features were selected according to F-tests.

The use of AdaBoost for classifier weight selection in most of the cases did not significantly improve the results compared to the majority and weighted voting ensemble. Due to the nature of AdaBoost, during the weight calculation process, the algorithm can reach optimal weights using only a few of the classifiers and ignore the rest, which can hinder the robustness of the ensemble.

Instead of focusing on the classification of feature types individually, combined features were also evaluated. Table 7 clearly shows the benefit of feature selection when compared to using all 162 features. For the most part, the classification results for features selected based on F-tests and ReliefF were higher than the baseline results for selecting all features. In addition, feature selection from all features gave promising results, especially, while using only the top-ranked features based on ReliefF. Features used in Table 7 (last row) were selected according to the same procedure used for feature types.

## 7. Conclusions

This study showed the results for linear (RBP, APV, SASI) and nonlinear (HDF, LZC, DFA) EEG features in various combinations for classification of long-lasting effects of depression.

The described feature types and classification methods (RBF SVM, LDA, NB, kNN, D3) were used to classify 20 age- and gender-matched subjects. The 10 healthy and 10 subjects who had depression were classified with 82.55% accuracy with the HDF using D3 and 80.70% with the LZC using the RBF SVM binary classifier. The results improved when the algorithms such as univariate feature ranking using F-tests and ReliefF were used, which improved the classification accuracy up to 91.5%. In addition, the ensemble setup with a majority voting reached 93.30% using the NB classifier. The results also suggest that electrodes A_rbp_.O1, A_rbp_.O2, and B_rbp_.O2 selected from all available features according to ReliefF were sufficient to classify the subjects with 80–95% accuracy. The best combination, which achieved significantly high accuracy among all classifiers, was an ensemble using ReliefF-selected features with equally weighted predictions for all feature types. The study shows that EEG features used in classifying patients with depression at the time of the recording can also be used to measure and classify the long-lasting effects of depression.

The obtained results give reasonable justification for further gathering of EEG data according to the currently used protocol to measure the long-lasting effects of depression. As future work, we are planning to raise funding for a large-scale study and further test the proposed approach with the aim of using it in assisted diagnostics.

## Figures and Tables

**Table 1:** Information about subjects in the dataset.

			EST-Q				
Sex	Age	HAM-D >7	DEP >11	AUR >11	PAF >6	SAR >3	AST >6
M	24/25	8/7	13/5	0/7	0/0	2/0	5/7
F	34/33	9/1	5/1	12/3	0/0	0/0	5/2
F	35/35	21/6	16/7	11/8	0/2	2/1	10/4
M	35/36	4/1	4/4	3/8	1/0	0/1	0/3
F	37/35	13/3	8/4	11/6	0/0	0/1	13/2
F	38/39	9/0	4/3	17/7	2/1	0/0	11/3
M	43/40	6/1	8/3	9/2	0/0	1/0	7/2
F	46/46	12/5	8/6	2/3	0/0	1/0	3/1
F	48/48	8/0	4/2	7/8	0/1	2/0	6/5
M	53/60	3/7	11/5	8/8	0/0	0/1	8/9

Subject pairs: depressed/healthy.

**Table 2:** Baseline classifier accuracy for all feature types.

	Classifier Accuracy (%)				
Feature Type	RBF SVM	LDA	Naive Bayes	kNN	D3
T_rbp_	54.40	65.00	73.30	44.45	38.65
A_rbp_	50.00	64.15	70.95	58.70	66.55
B_rbp_	70.00	52.90	65.05	62.20	79.85
G_rbp_	38.45	52.95	59.40	50.90	54.85
APV	35.40	27.05	31.85	37.70	64.65
SASI	54.55	55.00	54.60	59.10	53.15
HFD	55.40	41.55	51.80	71.55	82.55
LZC	80.70	57.10	58.50	75.95	63.50
DFA	68.15	75.25	63.15	70.80	74.55

**Table 3:** Selected electrodes based on the F-test and ReliefF. Electrodes are ordered according to their importance score.

	Selected Features	
Feature Type	Univariate Feature Ranking Using F-Tests	ReliefF
T_rbp_	F4 F8	O1 PZ P4 C4 P3
A_rbp_	F7 C3 T5 PZ P4 T6 O1 O2	O1 O2
B_rbp_	O1 O2 F4	O2 O1
G_rbp_	F7 F3 FZ F4	FP1
APV	T3 C3 F3	F3
SASI	FP1 FP2 F7 F3	FP1 F3
HFD	FP1 FP2 FZ F8 C3 T5 PZ O1 O2 F7 F3	FP1 O1
LZC	F3 F4 T4 FP1 FP2 FZ F8 P3 PZ O1 O2 F7 C3 C4 T5 P4	FP1 FZ
DFA	O1 O2 FP2 F7 P3 PZ FP1	FP1 FP2 O1

**Table 4:** Classifier accuracy for features selected by univariate feature ranking using F-tests.

	Classifier Accuracy (%)				
Feature Type *	RBF SVM	LDA	Naive Bayes	kNN	D3
T_rbp_ (2)	55.20	65.60	69.85	55.75	45.70
A_rbp_ (8)	62.35	73.85	70.95	72.65	67.15
B_rbp_ (3)	90.00	79.90	82.05	91.50	85.85
G_rbp_ (4)	70.20	52.20	71.10	54.55	60.00
APV (3)	61.00	50.25	47.75	55.55	62.95
SASI (4)	60.55	65.40	61.45	52.65	66.95
HFD (11)	73.40	42.20	53.20	75.70	82.55
LZC (16)	74.90	58.40	59.45	75.00	68.15
DFA (7)	73.05	50.35	55.45	75.85	68.85

(*) Number of features used (see Table 3).

**Table 5:** Classifier accuracy for features selected by the ReliefF algorithm.

	Classifier Accuracy (%)				
Feature Type *	RBF SVM	LDA	Naive Bayes	kNN	D3
T_rbp_ (5)	66.15	79.60	80.00	72.25	55.85
A_rbp_ (2)	81.20	78.70	75.95	90.00	85.30
B_rbp_ (2)	90.00	80.85	79.90	90.00	90.00
G_rbp_ (1)	75.00	69.00	75.00	70.00	63.25
APV (1)	62.85	37.50	62.80	71.20	66.40
SASI (2)	63.35	72.35	70.70	55.00	72.55
HFD (2)	77.45	53.65	66.20	81.00	85.70
LZC (2)	81.25	78.25	72.75	81.95	69.55
DFA (3)	78.80	56.40	72.55	86.00	72.90

(*) Number of features used (see Table 3).

**Table 6:** Ensemble classifier accuracy.

	Classifier Accuracy (%)				
Features and Ensemble Type	RBF SVM	LDA	Naive Bayes	kNN	D3
All + Maj.	70.55	48.85	61.85	68.65	79.05
F-test + Maj.	80.80	65.60	79.55	77.85	75.70
ReliefF + Maj.	88.30	80.85	93.30	88.25	88.25
All + Weig.	65.50	51.20	63.85	69.00	73.80
F-test + Weig.	79.90	69.45	77.25	78.85	73.80
ReliefF + Weig.	84.70	75.65	83.15	88.95	85.55
All + Ada.	70.80	53.10	71.40	63.75	69.15
F-test + Ada.	79.20	62.95	81.85	84.70	70.50
ReliefF + Ada.	81.05	72.20	78.50	86.70	79.85

Note: F-tests and ReliefF features are from Table 3.

**Table 7:** Classifier accuracy for concatenated features.

	Classifier Accuracy (%)				
Features	RBF SVM	LDA	Naive Bayes	kNN	D3
All features	53.25	52.35	65.20	55.50	56.70
F-test Table 3	56.70	62.10	80.75	72.55	72.90
ReliefF Table 3	70.00	71.75	76.90	80.00	65.05
F-test: top 44 features	64.60	69.10	77.00	81.25	74.40
ReliefF **	89.25	80.00	81.80	95.00	95.00

** Top ranked: A_rbp_.O1, B_rbp_.O2, A_rbp_.O2.

## Data Availability

Information about obtaining the dataset can be requested by contacting M. Bachmann at maie.bachmann@taltech.ee.
